# Novel hypothesis on the origin of gastroschisis?

**DOI:** 10.1007/s00383-022-05290-0

**Published:** 2022-12-09

**Authors:** Carmen Mesas Burgos

**Affiliations:** Department of Pediatric Surgery, Karolinska Institute, Stockholm, Sweden

**Keywords:** Gastroschisis, Ethiology, Congenital abdominal wall defects

In this number, the article of Tanaka and Oshio propose a novel hypothesis on the origin of gastroschisis, based on clinical observations. According to the authors, gastroschisis might develop due to dehiscence at the junction of the umbilical cord and the umbilical skin at the root of the umbilical cord. At this level, the authors divided the umbilical skin into two different anatomical structures, the ‘transitional zone’, closest to the cord, and the ‘skin’ zone. Partial dehiscence occurring at the umbilical skin, the weakest abdominal site vulnerable to increased intraabdominal pressure, at the level of the “transitional zone” at the junction of the umbilical cord could give arise to gastroschisis.

Several theories on the development of gastroschisis have been postulated over the years: from an early abnormal body wall formation, to vascular involvement, or yolk sac implication. Involvement of the cord and common developmental pathogenesis with Omphalocele has also been discussed for decades. Already in 1953, Moore proposed distortion of myotomes migration proposed [1], while Duhamel in 1963 postulated a failure of mesenchyme differentiation within the lateral fold and subsequent resorption of the ectoblastic layer, leaving an uncovered defect [2]. Other theories have assumed a vascular disruption of the right omphalomesenteric artery, that would cause infarction of the cord base and surrounding abdominal wall. De Vries proposed in 1980 that an early involution of the right umbilical vein would weaken the somatopleure and allow its rupture along the body stalk junction [3]. More recent theories do not involve the umbilicus in the origin of gastroschisis. Feldkamp in 2007 suggested that abnormal lateral folding of the embryo would prevent the normal merging of the yolk stalk within the cord [4]. The assumption that the cord is not involved in gastroschisis relies on the observation of a thin skin bridge between the cord and the defect, already described by Shaw in 1975 [5]. Bargy described gastroschisis in 2014 as a physiological hernia located in the right side of an otherwise tight cord, through what they described as “pars flacida” [6], that reminds on the “transitional zone” proposed in this article.

Tanaka and Oshio describe in this article a wider ‘transitional zone” on the right side of the umbilical cord than on the left due to vanishing structures explaining the evisceration of gastroschisis that is likely to occur on the right side, and postulate that this ‘transitional zone’ has potential to shrink, resulting in vanishing or closed gastroschisis but also explaining the successful treatment of gastroschisis with ‘suture-less closure’ [7].

Thus, this new theory proposes a dehiscence at the junction of the umbilical cord and umbilical skin as a pathogenetic mechanism for gastroschisis, without involvement of the cord.

The causal agents for this intrauterine event to occur are yet to be determined. Animal models and early embryological studies will be required to address this question effectively.
